# Impact of Electron Transport Layers on Hysteresis and Performance of Ambient‐Processed Perovskite Solar Cells

**DOI:** 10.1002/cssc.202502742

**Published:** 2026-04-15

**Authors:** Qian Chen, Abhinav K. Singh, Hissah Alghathami, Hongbo Mo, Feihong Li, Richard J. Curry, Laurie J. Phillips, Amanda J. Hughes

**Affiliations:** ^1^ Department of Materials, Design & Manufacturing Engineering School of Engineering University of Liverpool Liverpool UK; ^2^ Stephenson Institute for Renewable Energy Department of Physics University of Liverpool Liverpool UK; ^3^ Department of Materials School of Natural Sciences The University of Manchester Manchester UK; ^4^ Department of Electrical and Electronic Engineering Photon Science Institute University of Manchester Manchester UK; ^5^ Department of Electrical and Electronic Engineering University of Manchester Manchester UK

## Abstract

Ambient processing of perovskite solar cells (PSCs) offers a promising route to scalable and low‐cost manufacturing. While substantial progress has been achieved in improving power conversion efficiency (PCE), the hysteresis behavior of ambient‐processed devices remains insufficiently understood. This study examines hysteresis in PSCs fabricated in ambient air at 40–65% Relative humidity (RH) using multiple absorber compositions, including MAPbI_3_, CsFAPbI_3_, and Cs_2_AgBiBr_6_. Severe hysteresis is observed in devices employing planar TiO_2_ or SnO_2_ electron transport layers (ETLs), attributed to amplified moisture‐ and oxygen‐induced defect formation in ambient air. To overcome this challenge, ETL architecture is systematically engineered by adjusting planar TiO_2_ thickness and incorporating mesoscopic TiO_2_ architecture with controlled thicknesses. An optimized configuration featuring an approximately 140 nm mesoporous layer substantially reduces hysteresis, lowering the hysteresis index (HI) in MAPbI_3_ PSCs from 0.52 for planar TiO_2_ to 0.19, enhancing stability while maintaining high PCE. Similar improvements are demonstrated for CsFAPbI_3_, where the HI decreases from 0.56 for planar TiO_2_ and 0.47 for planar SnO_2_ to 0.38, and for Cs_2_AgBiBr_6_, where the HI decreases from 0.32 to 0.08. These findings highlight ETL structural engineering as an effective strategy for mitigating hysteresis and enabling reliable ambient‐processed PSCs for scalable manufacturing.

## 1 Introduction

With the rapid advancement of perovskite solar cells (PSCs) and power conversion efficiencies (PCEs) exceeding 27%, the technology is steadily moving toward commercialization [1]. However, achieving high efficiency, stability, and reproducibility typically requires device fabrication under inert atmospheres, as perovskite film crystallization is highly sensitive to ambient factors such as humidity, oxygen, and solvent atmosphere [2, 3]. Most high‐performance PSCs reported to date have been fabricated under ultra‐dry conditions (typically < 0.1 ppm H_2_O and < 10 ppm O_2_) to promote high crystallinity and reduce defect densities in the perovskite films [4, 5, 6]. Nevertheless, for scalable and cost‐effective commercialization, it is crucial to develop low‐cost, simplified fabrication processes that enable the formation of high‐quality perovskite films under ambient conditions [2]. The ability to process perovskite films in air would greatly facilitate large‐scale manufacturing and significantly reduce production complexity and cost [3].

Achieving uniform and compact perovskite films under ambient conditions requires mitigating the adverse effects of moisture on film crystallization [7, 8]. During film deposition, perovskite precursor solutions typically containing hygroscopic solvents such as N,N‐Dimethylformamide (DMF) and Dimethyl sulfoxide (DMSO) readily absorb moisture from the surrounding atmosphere [7]. The increase in moisture content delays the onset of supersaturation, reduces nucleation density, and increases the critical nucleus radius [9]. These effects collectively promote island‐like or dendritic film morphologies after annealing, ultimately leading to poor photovoltaic performance of the PSCs [8].

To address this challenge, several strategies, including solvent engineering [10, 11], additive engineering [12], antisolvent treatment [7, 13], and air‐knife‐assisted deposition [3, 14], have been developed to produce smooth, compact, and highly crystalline perovskite films in ambient air, thereby enhancing PSC performance. Our recent studies have demonstrated a green antisolvent strategy based on ethyl acetate that enables the formation of uniform, compact perovskite films under ambient conditions. Using this approach, high‐quality lead‐based perovskites, including MAPbI_3_ and CsFAPbI_3_, were successfully fabricated in ambient air [15, 16, 17]. More recently, we further extended this approach to the deposition of high‐quality, lead‐free Cs_2_AgBiBr_6_ films under relatively high Relative humidity (RH) of 60–70%, highlighting a pathway toward more sustainable and low‐toxicity perovskite technologies [18]. These results demonstrate that high‐quality perovskite films and efficient PSCs can be realized under ambient conditions.

However, despite improvements in PCE that narrow the gap with the PSCs fabricated in gloveboxes or other controlled environments, the hysteresis behavior of ambient‐processed PSCs remains poorly understood. As hysteresis not only influences accurate efficiency measurement but also reflects interfacial and ionic processes linked to long‐term stability, understanding and mitigating hysteresis is essential for advancing ambient‐processed PSCs [19, 20]. While previous studies have demonstrated that modifying the electron transport layer (ETL) materials, thickness, or architecture can influence hysteresis behavior in PSCs fabricated under controlled environments, including devices processed in gloveboxes [21, 22, 23], the effectiveness of these strategies for devices fabricated under ambient conditions remains insufficiently explored.

In this study, we first fabricated PSCs with various compositions, including MAPbI_3_, CsFAPbI_3_, and Cs_2_AgBiBr_6_, under ambient air with a relative humidity of 40–65%, using planar device architectures based on either TiO_2_ or SnO_2_ ETLs. All planar devices exhibited pronounced hysteresis behavior, indicating severe interfacial charge accumulation and ion migration. To mitigate these effects, we selected MAPbI_3_ as a model system and systematically engineered the ETL by tuning the compact TiO_2_ thickness and introducing mesoscopic architectures with mesoporous TiO_2_ of varying thicknesses. Increasing the compact TiO_2_ thickness from 30 to 50 nm with a planar architecture reduced the hysteresis index (HI) from 0.70 to 0.52 and improved the PCE from 14.02% to 16.02%, attributable to enhanced interfacial contact and more efficient electron extraction. Further incorporation of a 140 nm mesoporous TiO_2_ scaffold significantly reduced the HI from 0.52 to 0.19 while maintaining a comparable PCE of 15.67%, demonstrating a favorable balance between hysteresis suppression and charge‐transport efficiency. Increasing the mesoporous layer thickness to 220 nm yielded an even lower hysteresis index of 0.09 at the cost of lower PCE due to increased series resistance and poor quality of the perovskite capping layer. The optimized Meso‐140 device also demonstrated improved stability, retaining over 85% of its initial PCE after aging for 28 days in ambient air with an RH of 40–60%, compared with 75% retention for planar compact TiO_2_ devices aged under the same conditions. Extending this mesoscopic ETL architecture to CsFAPbI_3_ and Cs_2_AgBiBr_6_ similarly yielded substantial hysteresis suppression, confirming the generality of this strategy. These findings establish an effective ETL engineering strategy that suppresses ionic accumulation, stabilizes charge transport, and enhances device reliability and stability, representing a promising pathway toward ambient processing of reliable and efficient PSCs.

## 2 Result and Discussion

**FIGURE 1:**
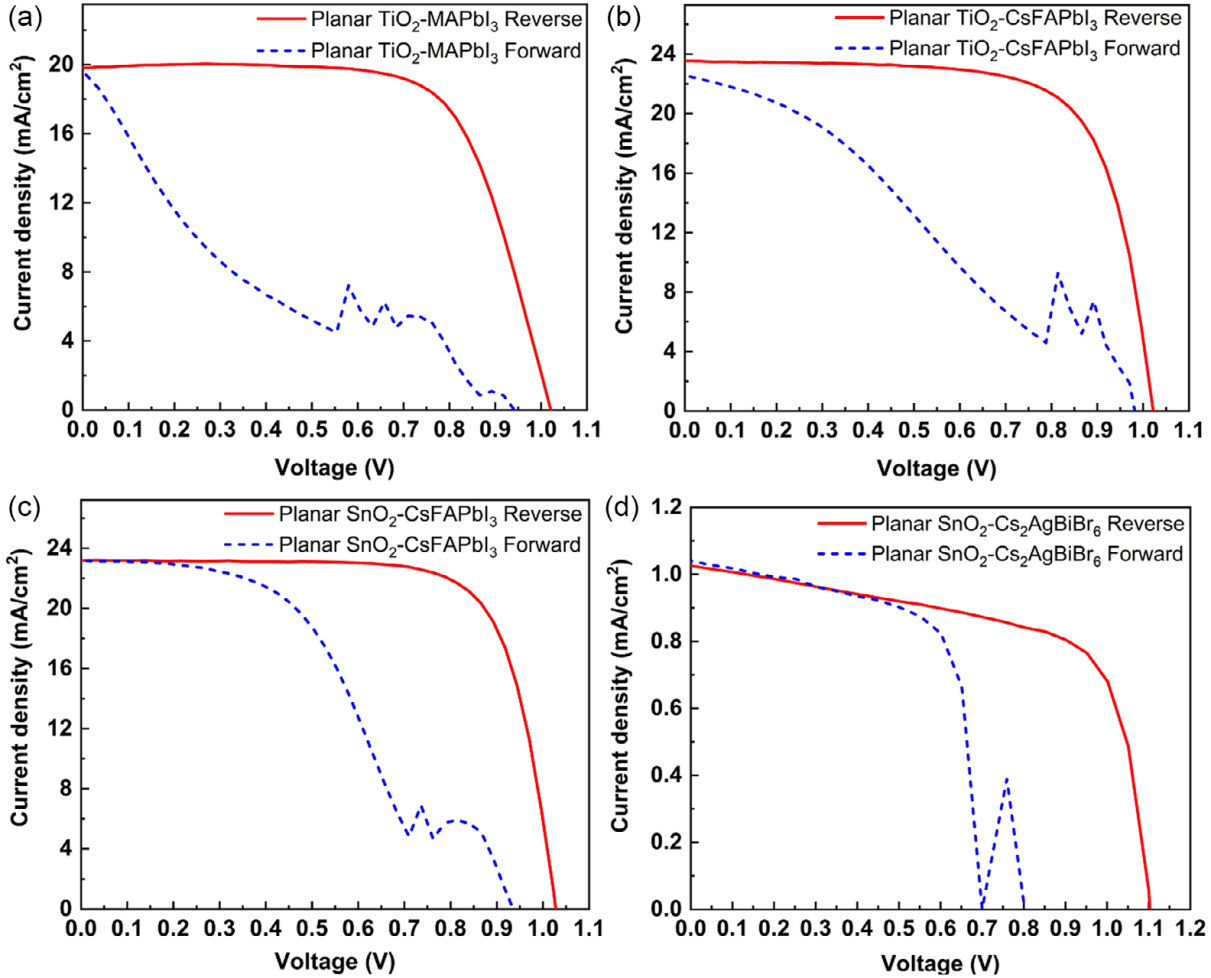
*J–V* characteristics of ambient‐processed PSCs based on the (a) planar TiO_2_‐MAPbI_3_, (b) planar TiO_2_‐CsFAPbI_3_, (c) planar SnO_2_‐CsFAPbI_3_, and (d) planar SnO_2_‐Cs_2_AgBiBr_6_ under reverse and forward voltage sweeps.

We first fabricated ambient‐processed PSCs with different perovskite compositions, including MAPbI_3_, CsFAPbI_3_, and Cs_2_AgBiBr_6_, under ambient air with a relative humidity of 40–65%, using planar device architectures based on compact TiO_2_ or SnO_2_ ETLs in an Indium tin oxide (ITO)/ETL/perovskite/Spiro‐OMeTAD/Au configuration. Detailed fabrication procedures are provided in Section 4. Figure 1 presents the corresponding current density–voltage (*J–V*) characteristics for the best‐performing devices. All ambient‐processed planar devices exhibited pronounced hysteresis, with substantially higher performance observed in the reverse scans compared with the forward scans. The photovoltaic parameters for both scan directions are summarized in Table S1, Supporting Information.

While the reverse scans appear smooth and well‐behaved, the forward scans display severely distorted current responses characterized by sharp fluctuations and spike‐like features, indicating unstable carrier extraction in ambient‐processed planar PSCs. To quantify the level of hysteresis in each sample, an HI is calculated, as shown in Equation (1) .

(1) $$\mathrm{HI}=\frac{{\mathrm{PCE}}_{\mathrm{RS}}-{\mathrm{PCE}}_{\mathrm{FS}}}{{\mathrm{PCE}}_{\mathrm{RS}}}\times 100\%$$

The ambient‐processed planar devices show HI values of approximately 0.32 to 0.70, as shown in Table S1.

Hysteresis in PSCs has been widely attributed to several dynamic processes, including ion migration, trap‐assisted recombination, ferroelectric and capacitive polarization, and interfacial band‐alignment asymmetry [24, 25]. Among these, ion migration and trap‐mediated recombination are significantly amplified in ambient‐processed perovskites, where moisture and oxygen increase vacancy populations and interfacial trap density [26]. These defects enhance ionic mobility and promote charge accumulation at ETL/perovskite interfaces. The resulting ionic charge partially screens the built‐in field and forces diffusion‐dominated carrier transport, leading to increased recombination and reduced fill factor (FF) and open‐circuit voltage (*V*_OC_) [27]. The S‐shaped distortions observed in *J–V* curves indicate interfacial transport barriers [28, 29]. During forward bias scanning, ionic redistribution and trap occupation evolve dynamically. Transient interfacial barriers can form and collapse, producing abrupt current transitions between voltage points [30, 31].

The strong asymmetry between scan directions can be explained by field stabilization under open‐circuit conditions. During the reverse scan, ions have reached a relatively equilibrated distribution that supports efficient field‐driven carrier extraction, which results in high PCE values. In contrast, during the forward scan, ongoing ionic redistribution progressively screens the internal field, leading to rapid collapse of performance and highly unstable current [25]. The similar hysteresis severity observed across all compositions indicates that ambient‐induced defect formation is the dominant factor rather than intrinsic differences among the perovskite absorbers. A glovebox‐processed CsFAPbI_3_ based on the planar SnO_2_ control sample exhibited a significantly reduced HI, as shown in Figure S1, Supporting Information, confirming that ambient‐induced defects primarily drive the observed hysteresis behavior. Notably, the ambient‐processed device delivers a reverse‐scan PCE of 17.76%, which is comparable to the efficiency of the glovebox‐processed control, highlighting that high performance can be achieved even under 40–60% relative humidity. However, the strong discrepancy between reverse and forward scans underscores the reverse‐scan values. This result further emphasizes the critical importance of suppressing hysteresis for reliable evaluation and practical deployment.

To address the hysteresis issue, strategies capable of suppressing interfacial ionic accumulation and enhancing electron extraction at the ETL/perovskite interface are required. We employed MAPbI_3_ as a model system to systematically examine the influence of ETL architecture on hysteresis by systematically tuning the compact TiO_2_ thickness and introducing mesoscopic designs incorporating mesoporous TiO_2_ layers of varying thickness.

**FIGURE 2:**
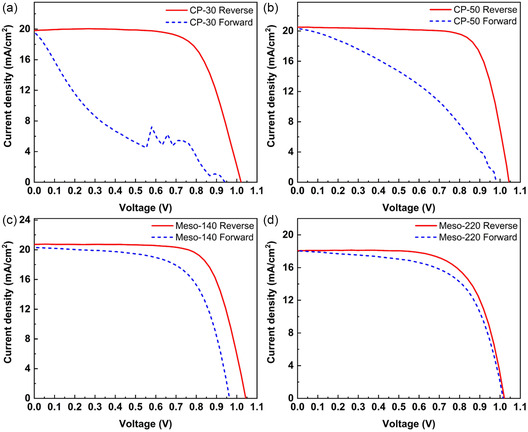
*J–V* characteristics of ambient‐processed MAPbI_3_ PSCs employing four different ETL architectures: (a) CP‐30, (b) CP‐50, (c) Meso‐140, and (d) Meso‐220 under reverse and forward voltage sweeps.

Figure 2 presents the corresponding typical *J–V* curves for four ETL configurations: a compact TiO_2_ layer of approximately 30 nm (CP‐30), a thicker compact layer of approximately 50 nm (CP‐50), a mesoscopic structure consisting of a 140 nm mesoporous TiO_2_ layer on top of a 50 nm compact layer (Meso‐140), and a thicker mesoporous layer of approximately 220 nm (Meso‐220). ETL thicknesses were determined by cross‐sectional scanning electron microscopy (SEM) analysis, as shown in Figure S2.

**FIGURE 3:**
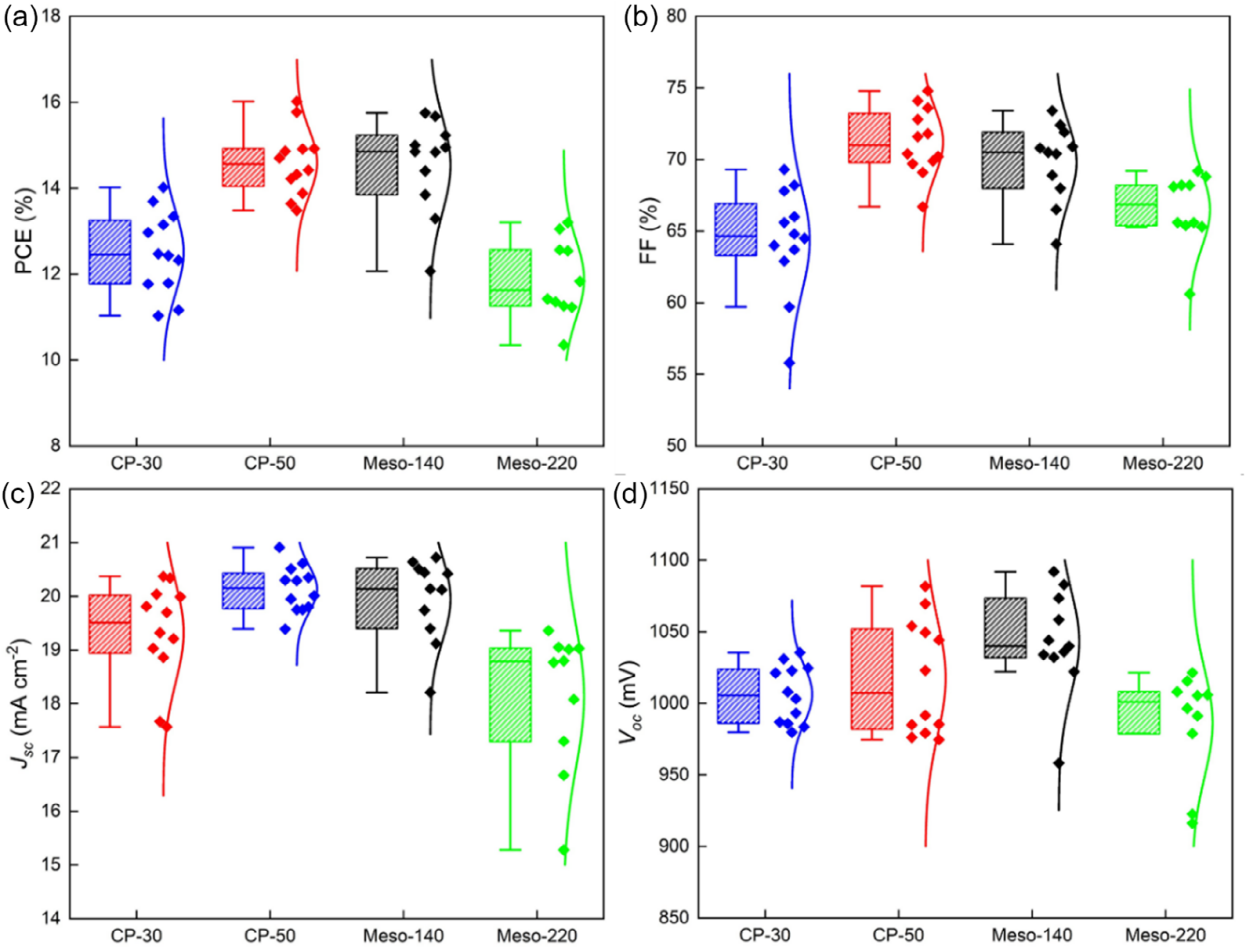
Photovoltaic parameter distribution of (a) PCE, (b) FF, (c) *J*_SC_, and (d) *V*_OC_ for the ambient‐processed MAPbI_3_ PSCs based on CP‐30, CP‐50, Meso‐140, and Meso‐220.

Increasing the compact TiO_2_ thickness from 30 to 50 nm yielded a noticeable reduction in hysteresis, with the HI decreasing from 0.70 to 0.52. Correspondingly, the severe spike‐like current fluctuations evident in the forward scan of the CP‐30 devices were substantially suppressed in CP‐50, indicating improved carrier extraction dynamics. The average PCE, calculated from more than 10 devices based on reverse scans, increased from 12.51% (champion PCE of 14.02%) for CP‐30 to 14.60% (champion PCE of 16.02%) for CP‐50, primarily due to an improvement in fill factor from 64.4% to 71.2%, as shown in Figure 3. The detailed photovoltaic parameters are summarized in Table S2.

A scan‐sequence dependence is also observed, as shown in Figure S3. When the reverse scan is performed first, the subsequent forward scan is smooth, whereas a forward‐first scan produces spike‐like features. This behavior reflects the different ionic initial conditions established by the first scan. Starting from open‐circuit conditions during a reverse‐first scan partially equilibrates ionic distributions and stabilizes the internal field, whereas a forward‐first scan begins from a nonequilibrated ionic configuration. As the bias approaches *V_oc_*, rapid ionic redistribution and trap occupation can transiently destabilize interfacial band alignment, producing abrupt current discontinuities. After one scan, the device becomes partially conditioned, and subsequent scans are smoother. Importantly, the reverse‐scan PCE, forward‐scan PCE, and hysteresis index remain similar regardless of scan order, indicating that the measurements were conducted within the quasi‐steady‐state regime and that the hysteresis originates from intrinsic device physics.

Introducing a mesoscopic structure significantly further reduced hysteresis. The Meso‐140 device exhibited a hysteresis index of 0.19 while maintaining an average PCE of 14.53% (champion PCE of 15.67%), similar to the CP‐50 device. The forward scan in Meso‐140 no longer showed current spikes and demonstrated a substantially higher FF of 64.8% compared with 38.7% for CP‐50, indicating more efficient charge extraction and suppressed interfacial charge accumulation. Increasing the mesoporous TiO_2_ thickness to 220 nm nearly eliminated hysteresis, reducing the HI to 0.09. However, the average PCE decreased to 11.88% (champion PCE of 12.56%), indicating a trade‐off between hysteresis suppression and overall photovoltaic performance. Devices incorporating the mesoporous TiO_2_ architecture show negligible scan‐sequence dependence and no current spikes (Figure S3), further indicating that the mesoscopic interface suppresses ionic accumulation and stabilizes interfacial electric fields.

To ensure that the observed hysteresis is not an artifact of measurement conditions, the measurement strategy was deliberately designed to ensure that the observed hysteresis originates from device physics and architecture. Recent studies have established that hysteresis evolves nonmonotonically with scan rate due to the competition between electronic transport and ionic migration dynamics [29, 32]. The low scan rates (<1 V s^−1^) allow ionic redistribution to approach quasi‐steady‐state conditions, enabling a more reliable assessment of operational hysteresis.

To confirm that the observed hysteresis is not an artifact of a single scan rate, we performed measurements at three low scan rates: 0.13, 0.25, and 0.43 V s^−1^. As shown in Figure S4a–c, ambient‐processed planar TiO_2_ devices based on the CP‐50 exhibit consistent hysteresis characteristics, with reverse‐scan PCE significantly exceeding forward‐scan PCE. As summarized in Figure S4d, the HI remains nearly constant across these scan rates, confirming that the pronounced hysteresis is an intrinsic device property rather than a scan‐rate artifact. We also observe that spike‐like current fluctuations become less pronounced at the slowest scan rate (0.13 V s^−1^). This behavior is consistent with dynamic trap filling and ionic redistribution processes. Slower voltage sweeping allows mobile ions and trapped carriers to redistribute more gradually, reducing transient field perturbations and stabilizing carrier extraction [31, 33].

On the other hand, Figure S5 shows that devices incorporating the mesoscopic TiO_2_ architecture exhibit substantially reduced hysteresis across all tested scan rates. The HI remains markedly lower than that of planar devices, and the forward–reverse PCE gap is minimal. Because both planar and mesoscopic devices were evaluated under identical quasi‐steady‐state scan conditions, these results confirm that hysteresis suppression arises from device architecture rather than scan‐rate selection.

Noticeable variations in *V*_OC_ and *J*_SC_, and in some cases both parameters, are observed between forward and reverse scans, particularly in ambient‐processed planar devices. Such behavior is characteristic of mixed ionic–electronic conductors and is commonly attributed to the redistribution of mobile ionic defects coupled with interfacial recombination processes [34, 35, 36].

Under illumination and applied bias, mobile ions can accumulate near transport‐layer interfaces, modifying the internal electric field and interfacial band bending. These electrostatic changes can reduce quasi‐Fermi level splitting and enhance nonradiative recombination, contributing to losses in *V*_OC_ [37]. Bias‐dependent ionic accumulation and trap‐mediated recombination can also distort interfacial energetics and reduce contact selectivity, impeding carrier extraction near short‐circuit conditions and lowering *J*_SC_ [36, 38]. Ionic space‐charge formation may further affect carrier transport and collection efficiency.

When ionic redistribution significantly perturbs interfacial energetics and the internal field, simultaneous reductions in *V*_OC_, *J*_SC_, and fill factor may occur, sometimes accompanied by S‐shaped distortions, consistent with ion‐induced transport barriers and increased recombination losses [37]. These scan‐dependent variations are often less pronounced in devices employing mesoporous TiO_2_ architectures, where the three‐dimensional interface distributes interfacial charge, facilitates electron extraction, and stabilizes band alignment, leading to more scan‐independent photovoltaic parameters [22].

**FIGURE 4:**
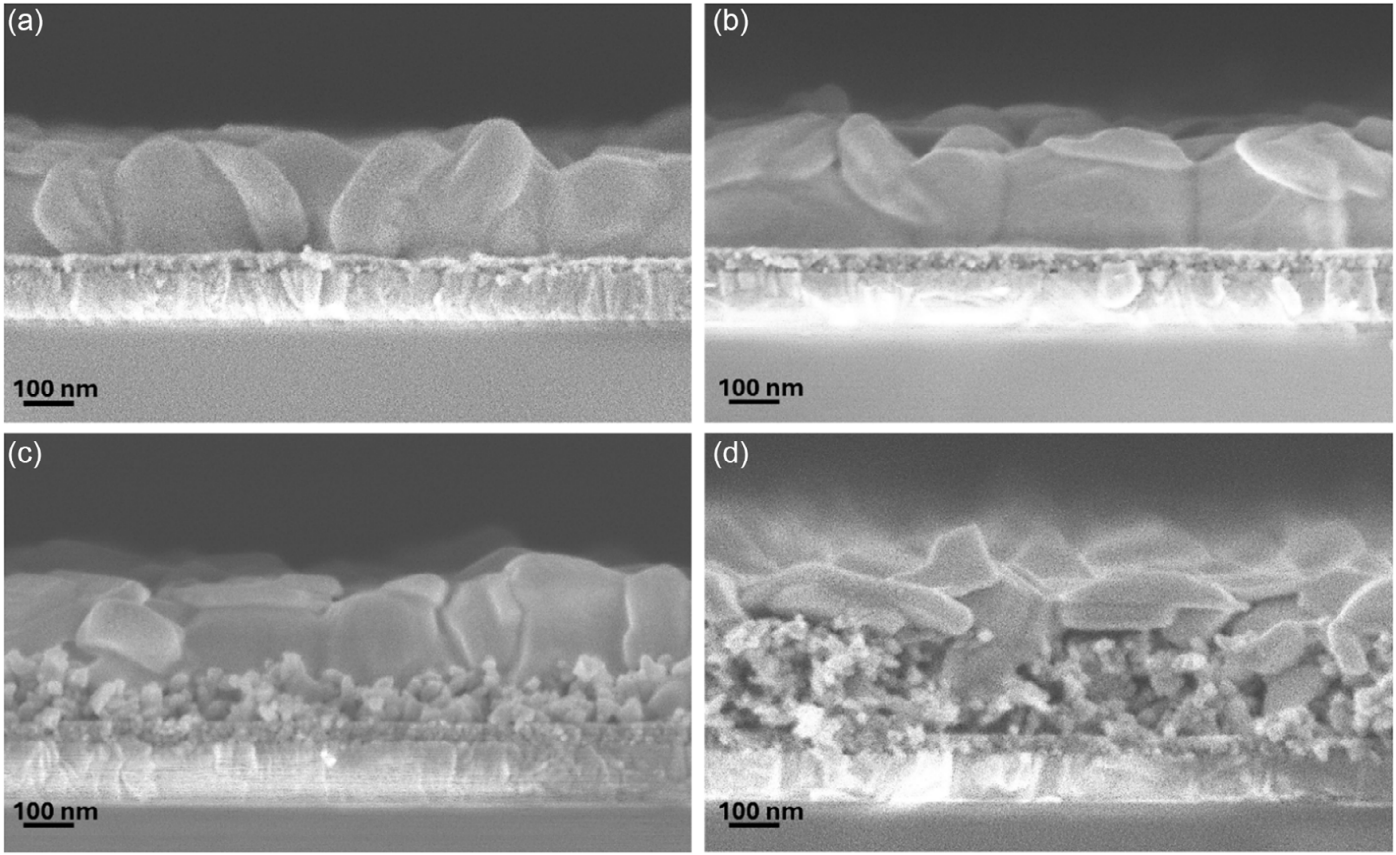
Cross‐sectional SEM view for the ambient‐processed MAPbI_3_ films based on the (a) CP‐30, (b) CP‐50, (c) Meso‐140, and (d) Meso‐220.

Cross‐sectional scanning electron microscopy (SEM) images of MAPbI_3_ films on different ETLs are shown in Figure 4. Both CP‐30 and CP‐50 devices exhibit smooth and pinhole‐free perovskite layers with thicknesses of approximately 240 nm. However, the CP‐30 device with a thinner TiO_2_ layer covering the ITO displays a rougher ETL/perovskite interface and less uniform contact as shown in Figure 4a, increasing the likelihood of direct perovskite–ITO interaction and associated trap formation. This likely contributes to the strong hysteresis and pronounced current fluctuations in CP‐30 due to enhanced interfacial recombination and ionic accumulation. The improvement for CP‐50 is attributed to enhanced interfacial electrical uniformity and reduced direct contact between perovskite and underlying ITO, as indicated by the more continuous and well‐defined interface, as shown in Figure 4b.

In contrast, the mesoscopic architecture in Meso‐140 (Figure 4c) enables perovskite infiltration throughout the mesoporous scaffold, forming confined nanocrystalline regions and a thinner but dense and uniform capping layer (~178 nm). This three‐dimensional interfacial contact provides an expanded charge–transfer surface area and shorter electron‐transport pathways, which reduce carrier residence time and suppress interfacial ionic buildup [22]. The distributed interface geometry also spreads electric‐field gradients throughout the porous network, reducing the driving force for localized clustering of mobile ions such as iodide vacancies or cations, thereby stabilizing the internal electric field during voltage sweeps [39]. As a result, ionic redistribution is minimized, eliminating the field collapse and current spike behavior seen in planar architectures. Increasing the mesoporous thickness to 220 nm (Figure 4d) results in a thicker infiltrated region and a significantly thinner (approximately 110 nm) capping layer with reduced grain continuity, which likely contributes to reduced PCE despite further hysteresis suppression.

**FIGURE 5:**
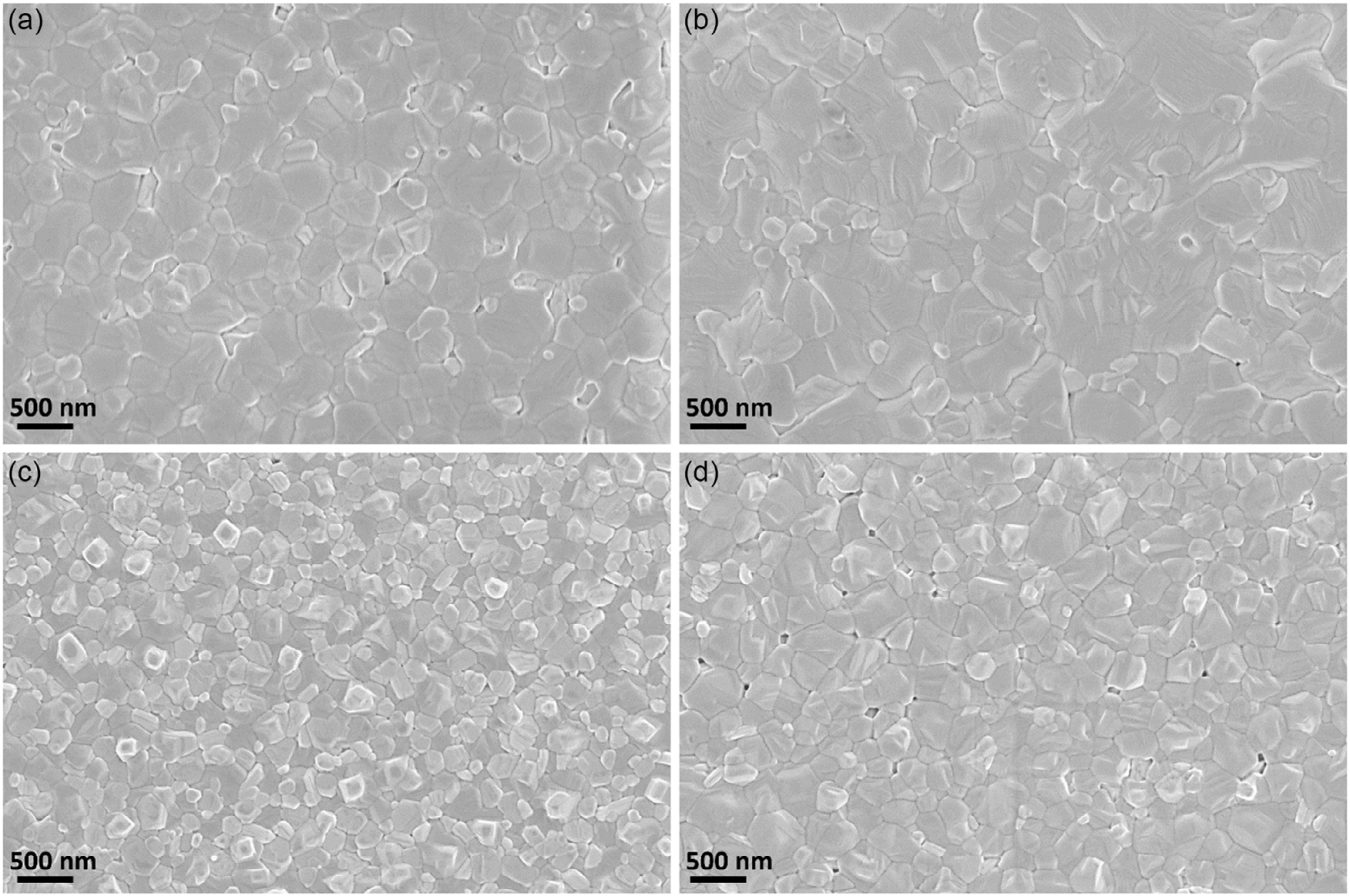
Top surface SEM images for the ambient‐processed MAPbI_3_ films based on the (a) CP‐30, (b) CP‐50, (c) Meso‐140, and (d) Meso‐220.

Surface SEM images in Figure 5 further support these conclusions. As shown in Figure 5a and b, CP‐30 and CP‐50 perovskite films exhibit larger surface grains of 0.16 and 0.19 μm^2^, while Meso‐140 (Figure 5c) and Meso‐220 (Figure 5d) films exhibit smaller average grain sizes of 0.06 μm^2^, consistent with the thinner capping layers observed from the cross‐sectional view in Figure 4. Average grain sizes were determined by analyzing more than 50 grains per sample from SEM images, as summarized in Figure S6. Meso‐140 film remains compact and has a uniform morphology, whereas Meso‐220 shows surface pinholes that could enable spiro‐OMeTAD infiltration and localized shunting, contributing to reduced *J*_sc_ and PCE.

**FIGURE 6:**
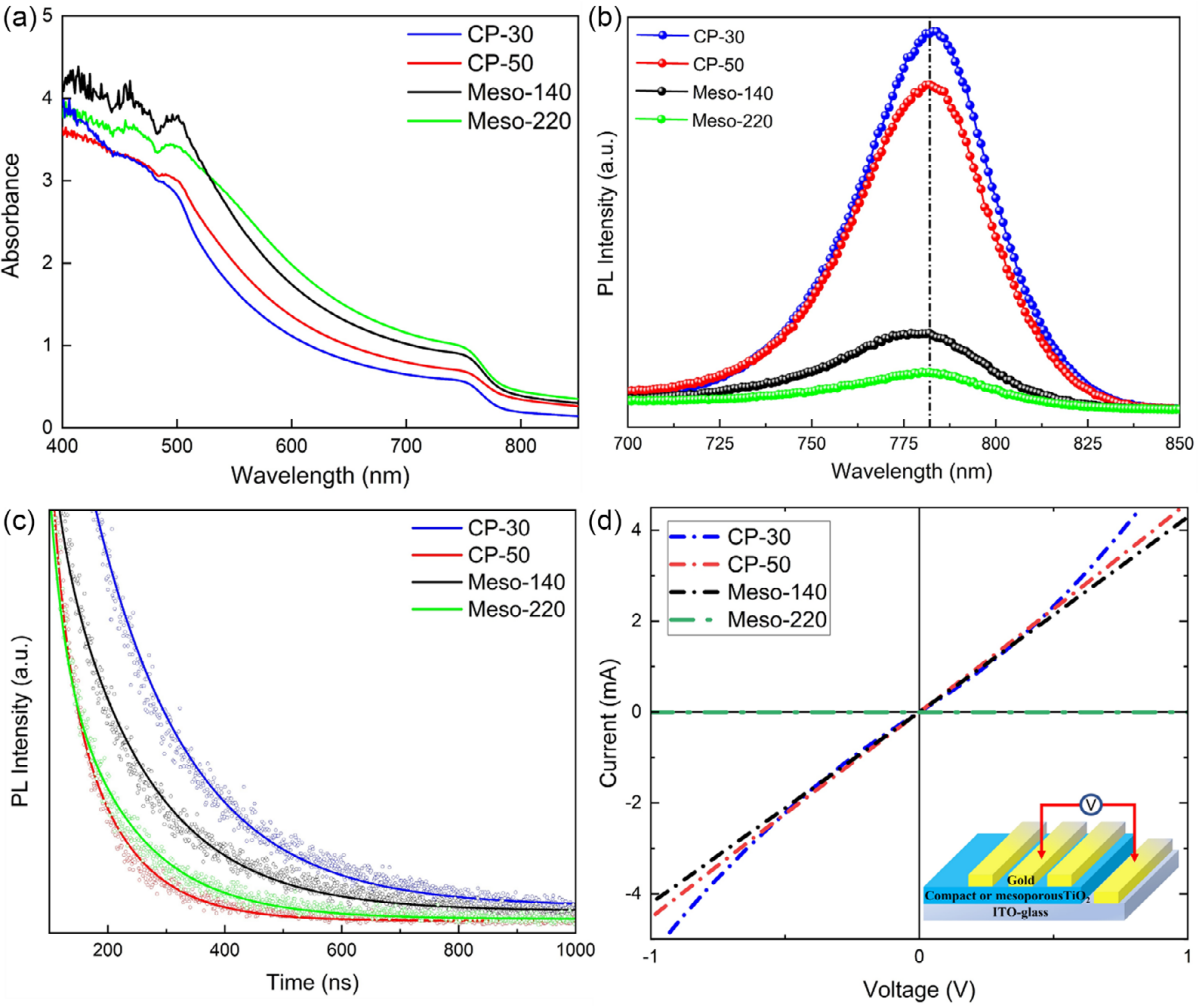
(a) Absorption spectra measured by UV–vis–NIR spectroscopy for the ambient‐processed MAPbI_3_ based on CP‐30, CP‐50, Meso‐140, and Meso‐220; (b) Steady‐state Photoluminescence (PL) spectra; and (c) Time‐resolved Photoluminescence (TRPL) spectra for the ambient‐processed MAPbI_3_ based on CP‐30, CP‐50, Meso‐140, and Meso‐220, (d) *I–V* curve for the CP‐30, CP‐50, Meso‐140, or Meso‐220 measured with an ITO/ETL/Au architecture.

Optical properties of the ambient‐processed MAPbI_3_ films deposited on different ETLs were examined using ultraviolet–visible (UV–vis) spectroscopy, as shown in Figure 6a. Both mesoscopic architectures (Meso‐140 and Meso‐220) exhibit stronger absorption in the range of 400–750 nm relative to the planar CP‐30 and CP‐50 devices, consistent with the presence of a thicker effective absorber layer composed of the perovskite‐infiltrated mesoporous TiO_2_ region and the upper capping layer. Notably, the Meso‐140 film displays slightly higher absorbance between 400 and 510 nm compared with Meso‐220. This difference is attributed to the denser and more uniform capping layer present in Meso‐140, whereas Meso‐220 contains a thinner and less continuous capping layer that decreases its optical density in the high‐energy region. This improved light harvesting in Meso‐140 correlates with its higher *J*_sc_ and overall superior photovoltaic performance compared with Meso‐220.

Steady‐state photoluminescence (PL) spectra in Figure 6b provide further insight into the interfacial carrier dynamics that govern hysteresis behavior. The CP‐30 sample exhibits the highest PL intensity, indicating inefficient electron extraction and long carrier lifetimes at the interface. Such prolonged carrier residence enhances the likelihood of ionic buildup and defect‐mediated nonradiative pathways, including iodide vacancy‐related traps, consistent with the severe hysteresis and current spike behavior observed in CP‐30. Increasing the compact TiO_2_ thickness to CP‐50 produces stronger PL quenching, reflecting improved interfacial charge transfer that reduces carrier accumulation and mitigates ionic vacancy formation, in agreement with its lower HI and improved FF.

The incorporation of a mesoporous TiO_2_ scaffold results in significantly enhanced PL quenching for Meso‐140, indicating substantially improved interfacial charge transfer. The perovskite infiltration, the enlarged interfacial contact area, and the shortened transport paths enable rapid electron extraction, decreasing the likelihood of local ionic screening of the internal field. This interpretation is supported by time‐resolved photoluminescence (TRPL) analysis (Figure 6c and Table S3), where Meso‐140 exhibits the shortest interfacial decay lifetime τ_1_ among the samples, demonstrating fast carrier transfer and reduced trap‐assisted recombination at the interface. Meanwhile, its moderate τ_2_ value indicates well‐preserved bulk crystallinity without excessive nonradiative recombination. In contrast, Meso‐220 displays the faster overall decay than Meso‐140 and the strongest PL quenching, yet the corresponding reduction in PCEs suggests considerable nonradiative recombination caused by poorer capping‐layer morphology.

Conductivity measurements using ITO/ETL/Au devices in Figure 6d show decreasing ETL sheet conductance with increasing TiO_2_ thickness, with Meso‐220 approaching near‐insulating behavior. This increased resistance for Meso‐220 likely contributes to its reduced photovoltaic performance despite minimal hysteresis. Overall, these results identify Meso‐140 as the most effective ETL configuration, offering an optimal balance between hysteresis suppression and photovoltaic performance.

**FIGURE 7:**
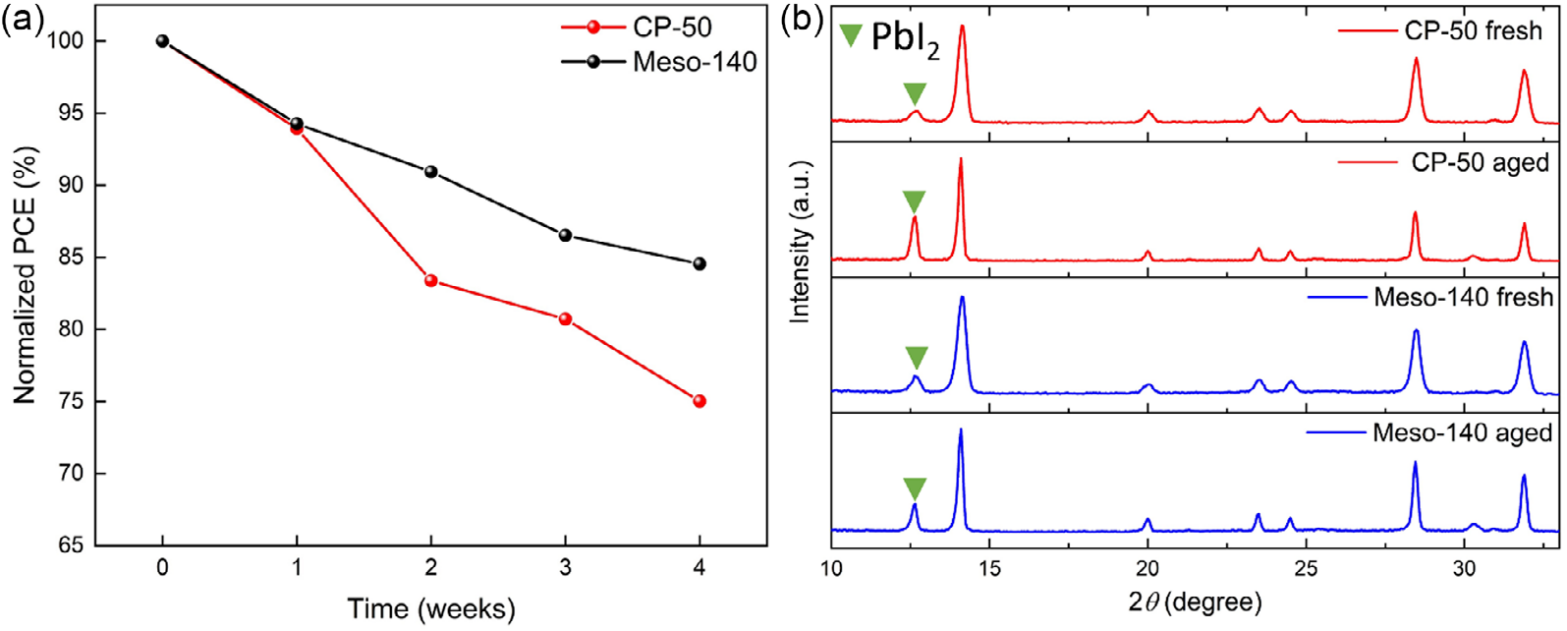
(a) Long‐term stability comparison of normalized PCEs for ambient‐processed MAPbI_3_ PSCs using CP‐50 and Meso‐140 ETL architectures aged in ambient air with an RH of 40–65%. (b) XRD patterns of fresh and aged ambient‐processed MAPbI_3_ films based on the CP‐50 and Meso‐140 ETL architectures.

Because hysteresis and ionic migration are directly linked to the long‐term stability of PSCs, we further examined device stability in unencapsulated ambient conditions (RH 40–65%). As shown in Figure 7a, devices based on the Meso‐140 architecture retained 85% of their initial PCE after 28 days of storage, yielding a final average PCE of 12.77% from an initial value of 15.10%. In contrast, devices based on CP‐50 retained only 75% of the initial PCE, decreasing from 15.20% to 11.40%.

To evaluate the impact of device degradation on hysteresis evolution, freshly prepared devices were compared with identical cells stored in ambient air at an RH of 50–70% for 9 days, as shown in Figure S7 and Table S4. Planar TiO_2_ devices (CP‐50) exhibit severe deterioration after 9 days, with the forward‐scan PCE decreasing from 2.84% to 0.61%, accompanied by a drastic fill factor reduction from 21.9% to 5.98%, while the reverse‐scan PCE decreases from 15.38% to 13.10%. This FF collapse is consistent with moisture‐ and oxygen‐induced degradation that increases trap‐assisted recombination and reduces contact selectivity, thereby strengthening ionic polarization and field screening at the charge‐selective interface [40, 41].

In contrast, devices incorporating the mesoscopic TiO_2_ scaffold (Meso‐140) exhibit improved stability. After aging, the forward‐scan PCE decreases from 5.59% to 2.51%, whereas the reverse‐scan PCE remains essentially unchanged from 14.93% to 15.03%. X‐ray diffraction (XRD) patterns in Figure 7b reveal a pronounced increase in the PbI_2_ decomposition peak at 12.7° for CP‐50/MAPbI_3_ film aged for 14 days, whereas aged Meso‐140/MAPbI_3_ film exhibits only a moderate increase, indicating reduced degradation. These observations support the correlation between hysteresis suppression and stability and suggest that the mesoporous structure can protect the infiltrated perovskite phase from moisture ingress while mechanically constraining the lattice and limiting mobile‐ion redistribution, thereby limiting interfacial deterioration.

**FIGURE 8:**
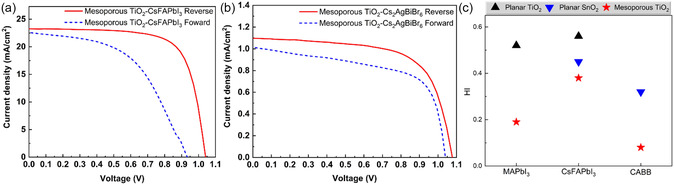
*J–V* characteristics of ambient‐processed PSCs based on the (a) Meso‐140‐CsFAPbI_3_ and (b) Meso‐140‐Cs_2_AgBiBr_6_ under reverse and forward voltage sweeps. (c) Hysteresis index comparison among ambient‐processed PSCs with different perovskite compositions based on various ETLs.

Encouraged by the performance of Meso‐140 with MAPbI_3_, we extended the architecture to ambient‐processed CsFAPbI_3_ and Cs_2_AgBiBr_6_ PSCs. As presented in Figure 8a and b, the spike‐like current fluctuations seen in planar TiO_2_ and SnO_2_ devices were eliminated, demonstrating improved charge extraction and reduced recombination and ionic accumulation. Figure 8c shows a substantial reduction in hysteresis index when employing the Meso‐140 architecture. The hysteresis index for CsFAPbI3 decreased from 0.56 for a planar TiO_2_ ETL and 0.47 for a SnO_2_ ETL to 0.38 using the Meso‐140 structure.

For Cs_2_AgBiBr_6_, the hysteresis index decreased from 0.32 for a planar SnO_2_ ETL to 0.08 using Meso‐140. In double perovskites such as Cs_2_AgBiBr_6_, strong electron–phonon coupling and polaronic carrier localization can limit carrier mobility and slow transport dynamics, increasing sensitivity to interfacial barriers and contact selectivity [42]. In planar architectures (Figure 1d), the ETL/perovskite interface constitutes a two‐dimensional extraction boundary where ambient‐processing‐induced defects, interfacial traps, and ionic accumulation can form space‐charge regions that impede carrier extraction. Such interfacial barriers reduce drift‐assisted collection near short‐circuit conditions, producing the slope and nonideal curvature in the low‐bias region. When the mesoporous TiO_2_ architecture is introduced (Figure 8b), the *J–V* curves become markedly flatter near 0 V, and hysteresis is strongly suppressed. The three‐dimensional interpenetrating interface reduces charge transport distance, distributes current pathways, and lowers interfacial resistance, thereby improving charge collection efficiency. Full photovoltaic parameters for both scan directions are summarized in Table S5. These results demonstrate that Meso‐140 maintains comparable reverse‐scan PCEs while universally suppressing hysteresis across different perovskite compositions, confirming the general applicability of this ETL engineering strategy.

## 3 Conclusions

In summary, we investigated the hysteresis behavior of ambient‐processed PSCs across multiple absorber compositions, including MAPbI_3_, CsFAPbI_3_, and Cs_2_AgBiBr_6_, fabricated under ambient air with an RH of 40–60%. All planar devices exhibited severe hysteresis, which arises from moisture and oxygen‐induced defect formation in ambient air, increased vacancy concentration, and significant interfacial ionic accumulation. These effects are particularly detrimental for devices based on the planar electron transport layers, such as compact TiO_2_ and SnO_2_. To address these challenges, we selected ambient‐processed MAPbI_3_ as a model system and systematically examined the influence of electron transport layer architecture on hysteresis by tuning the compact TiO_2_ thickness and introducing mesoscopic designs incorporating mesoporous TiO_2_ scaffolds with controlled thickness. Through this approach, we identified the Meso‐140 configuration as providing an optimal balance between high PCE, strong suppression of hysteresis, and improved long‐term stability when compared with devices based on planar compact TiO_2_. Extending this Meso‐140 ETL architecture to CsFAPbI_3_ and Cs_2_AgBiBr_6_ devices resulted in similarly effective hysteresis reduction, confirming the generality of this strategy across different perovskite compositions. These findings demonstrate that structural optimization of the ETL plays a critical role in mitigating ionic accumulation, stabilizing charge transport, and improving long‐term stability in ambient‐processed PSCs. Future progress should focus not only on improving efficiency but also on suppressing hysteresis through device architectural engineering and interfacial passivation methods in order to achieve reliable and scalable ambient fabrication of PSCs.

## 4 Experimental Section

### 4.1 Materials

All chemicals were used as received without additional purification. Patterned and unpatterned ITO–glass substrates, quartz glass slides, formamidinium iodide (FAI, 98%), and spiro‐OMeTAD (99%, unsublimed) were obtained from Ossila. Methylammonium iodide (MAI, 98%), lead chloride (PbCl_2_, 99.999%), cesium iodide (CsI, 99.999%), Hellmanex III detergent, ethyl acetate (EA, 99.8%, anhydrous), dimethylformamide (DMF, 99.8%), dimethyl sulfoxide (DMSO, 99.8%), chlorobenzene (99.9%), 4‐tert‐butylpyridine (4‐tBP, 98%), titanium diisopropoxide bis(acetylacetonate) (TTDP, 75 wt% in isopropanol), 1‐butanol, acetonitrile (99.8%), and lithium bis(trifluoromethanesulfonyl)imide (LiTFSI, 99.95%) were purchased from Merck. Silver bromide (AgBr, 99.5%), cesium bromide (CsBr, 99.9%), bismuth(III) bromide (BiBr_3_, 99%), lead iodide (PbI_2_, 99.9985%), and an aqueous SnO_2_ colloidal dispersion (tin oxide, 15 wt% in H_2_O) were purchased from Thermo Fisher Scientific. Mesoporous TiO_2_ paste (18NR‐T) was purchased from Greatcell Solar Materials.

### 4.2 Preparation of Electron Transport Layers (ETLs)

#### 4.2.1 Planar TiO_2_

ITO‐coated substrates were sequentially cleaned in 2% Hellmanex solution, deionized water, and ethanol, rinsed with water, and treated under UV–ozone for 15 min. Compact TiO_2_ films were deposited using TTDP in 1‐butanol. For the CP‐30 TiO_2_ layer, a 0.1 M TTDP solution was spin‐coated at 2000 rpm for 40 s, followed by drying at 100°C for 10 min in air and annealing at 450°C for 30 min. To form the thicker CP‐50 layer, the same procedure was repeated using an additional 0.3 M TTDP coating before final annealing.

#### 4.2.2 Planar SnO_2_

ITO substrates were cleaned and UV–ozone treated following the same procedure as above. The commercial SnO_2_ colloid was diluted with deionized water at a 1:4 volumetric ratio. The diluted dispersion (90 μL) was deposited onto the ITO by spin‐coating at 3000 rpm for 30 s and annealed on a hot plate at 150°C for 30 min to yield a uniform SnO_2_ layer.

#### 4.2.3 Mesoscopic TiO_2_

Mesoscopic ETLs were prepared by first forming a compact TiO_2_ underlayer, followed by deposition of a mesoporous TiO_2_ scaffold.

For both mesoscopic configurations, the compact layer was deposited using a 0.1 M TTDP solution (2000 rpm, 40 s, 100°C drying) followed by a second coating of 0.3 M TTDP using the same deposition and drying steps.

To fabricate the Meso‐140 structure, the mesoporous TiO_2_ layer was formed by spin‐coating a diluted 18NR‐T paste (1:5 weight ratio in ethanol) at 4000 rpm for 30 s, followed by annealing at 450°C for 30 min.

For Meso‐220, the same procedure was used except that the TiO_2_ paste was diluted at a 1:3 weight ratio in ethanol prior to spin‐coating, producing a thicker mesoporous scaffold after sintering at 450°C for 30 min.

### 4.3 Preparation of Perovskite Precursors, Films, and Devices

#### 4.3.1 MAPbI_3_ Precursor and Film Deposition

MAPbI_3_ precursor solutions were prepared by dissolving PbI_2_ and PbCl_2_ (460 and 28 mg, respectively) in a mixture of DMSO (0.15 ml) and DMF (0.8 ml). After stirring at 70°C for 30 min to complete dissolution, 190 mg MAI was added to the mixture, and the mixture was stirred for an additional 20 min to complete the precursor preparation. Prior to deposition, ETL‐coated ITO substrates were treated with UV–ozone for 15 min and then preheated at 70°C for 10 min. A volume of 90 µL of precursor solution was dispensed onto the warm substrate and spin‐coated at 4000 rpm for 30 s. Ethyl acetate (200 µL) was dripped onto the spinning film between 6 and 8 s to induce rapid crystallization. The coated substrates were then annealed at 120°C for 10 min to complete film formation.

#### 4.3.2 CsFAPbI_3_ Precursor and Film Deposition

The Cs_0_._1_FA_0_._9_PbI_3_ precursor was prepared by dissolving PbI_2_ (460 mg) in a mixture of 200 µL DMSO and 800 µL DMF at 70°C with continuous stirring for 1 h. After cooling, stoichiometric quantities of FAI (155 mg) and CsI (26 mg) were added and stirred overnight at room temperature to obtain a clear solution. For film fabrication, 90 µL of precursor was dispensed onto preheated ETL‐coated substrates and spin‐coated using a two‐step program of 1000 rpm for 10 s followed by 4000 rpm for 30 s. Ethyl acetate (200 µL) was added during the final 10 s of spinning. The intermediate film was then annealed at 100°C for 10 min to produce the crystalline perovskite layer.

#### 4.3.3 Cs_2_AgBiBr_6_ Precursor and Film Deposition

Cs_2_AgBiBr_6_ precursor solutions were prepared by dissolving CsBr (213 mg), AgBr (94 mg), and BiBr_3_ (224 mg) in 1 mL of DMSO, followed by vigorous stirring at 70°C for 1 h until a clear solution was obtained. The precursor was filtered through a 0.45 µm Polyethersulfone (PES) membrane immediately prior to use. ETL‐coated substrates were exposed to UV–ozone for 15 min and then preheated to 75°C for 10 min. Approximately 80 µL of precursor solution was spin‐coated at 4000 rpm for 30 s, during which 200 µL of ethyl acetate was dispensed between 7 and 8 s to promote rapid nucleation. Films were annealed at 280°C for 5 min to complete crystallization.

#### 4.3.4 Perovskite Solar Cell Fabrication

All processing steps for ambient‐fabricated devices were performed in laboratory air with a relative humidity of 40–65%, except for gold electrode deposition. After ETL and perovskite deposition, the hole‐transport layer was prepared by dissolving spiro‐OMeTAD (43 mg) in 0.5 mL chlorobenzene, followed by the addition of LiTFSI (10 µL of a 520 mg mL^−1^ stock in acetonitrile) and 4‐tBP (15 µL). The solution was spin‐coated onto the cooled perovskite layers. Gold electrodes (~90 nm) were deposited by thermal evaporation to complete the devices.

Reference devices fabricated in a glovebox followed identical procedures but were processed under a controlled atmosphere (H_2_O < 1 ppm, O_2_ < 10 ppm).

### 4.4 Characterization

Surface and cross‐sectional morphologies were examined using Zeiss Sigma and Zeiss Gemini field‐emission SEM. XRD patterns were collected in grazing‐incidence mode using a PANalytical XRD2 diffractometer. Optical absorption and transmission spectra were measured using a Shimadzu UV‐2401 PC spectrophotometer. Steady‐state PL was recorded using an Edinburgh Instruments FLS980 spectrometer, and time‐resolved PL (TRPL) was measured on an FLS1000 system using a 475 nm pulsed excitation source over a 1 µs time window. Current–voltage characteristics for PSCs were measured using either a Keithley 2400 source meter and an Ossila test board under simulated AM1.5G illumination (100 mW cm^−2^) from a class AAA Oriel solar simulator or a class AAA Ossila LED solar simulator connected to an Ossila test board. For all measurements, a 0.024 cm^2^ metal aperture was used to define the illuminated area, and the light intensity was calibrated using a certified quartz‐filtered silicon reference cell (Newport).

## Supporting Information

Additional supporting information can be found online in the Supporting Information section. **Supporting** **Figure S1**: *J–V* characteristics of glovebox‐processed PSCs based on the planar SnO_2_‐CsFAPbI_3_. **Supporting** **Figure S2**: Cross‐sectional SEM view of a) CP‐30, b) CP‐50, c) Meso‐140 and d) Meso‐220. **Supporting** **Figure S3**: *J–V* characteristics of ambient‐processed MAPbI_3_ PSCs based on the (a) CP‐50 with reverse scan first, (b) CP‐50 with forward scan first, (c) Meso‐140 with reverse scan first, and (d) Meso‐140 with forward scan first. **Supporting** **Figure S4**: *J–V* characteristics of ambient‐processed MAPbI_3_ PSCs based on the CP‐50 at the scan rates of (a) 130 mV s_‐1_, (b) 250 mV s_‐1_, and (c) 430 mV s_‐1_. (d) A summary of reverse‐scan PCEs, forward‐scan PCEs, and HI against various scan rates. **Supporting** **Figure S5**: *J–V* characteristics of ambient‐processed MAPbI_3_ PSCs based on the Meso‐220 at the scan rates of (a) 130 mV s_‐1_, (b) 250 mV s_‐1_, and (c) 430 mV s_‐1_. (d) A summary of reverse‐scan PCEs, forward‐scan PCEs, and HI against various scan rates. **Supporting** **Figure S6**: Grain size distribution for ambient‐processed MAPbI_3_ films based on the a) CP‐30, b) CP‐50, c) Meso‐140, and d) Meso‐220. **Supporting** **Figure S7**: *J–V* characteristics of ambient‐processed MAPbI_3_ PSCs based on the (a) CP‐50 fresh device, (b) CP‐50 aged in ambient air for 9 days (c) Meso‐140 fresh device, and (d) Meso‐140 aged in ambient air for 9 days. **Supporting** **Table S1**: Summary of photovoltaic parameters of the ambient‐processed planar PSCs based on the TiO_2_‐MAPbI_3_, TiO_2_‐CsFAPbI_3_, SnO_2_‐CsFAPbI3, and SnO_2_‐Cs_2_AgBiBr_6_. **Supporting** **Table S2**: Summary of photovoltaic parameters of the ambient‐processed MAPbI_3_ PSCs based on the CP‐30, CP‐50, Meso‐140, and Meso‐220. **Supporting** **Table S3** :Summary of TRPL results for the ambient‐processed MAPbI_3_ based on the CP‐30, CP‐50, Meso‐140, and Meso‐220. **Supporting** **Table S4**: Summary of photovoltaic parameters of the fresh and aged ambient‐processed PSCs devices based on the CP‐50 and Meso‐140. **Supporting** **Table S5**: Summary of photovoltaic parameters of the ambient‐processed CsFAPbI3 and Cs_2_AgBiBr_6_ PSCs based on the different ETLs.

## Funding

This work was supported by the Engineering and Physical Sciences Research Council (EP/X03660X/1 and EP/V008188/1).

## Conflicts of Interest

The authors declare no conflicts of interest.

## Supporting information

Supplementary Material

## Data Availability

The data that support the findings of this study are available from the corresponding author upon reasonable request.
