# Supplementary material for: Impact of Electron Transport Layers on Hysteresis and Performance of Ambient‐Processed Perovskite Solar Cells
Source: ChemSusChem. 2026 Apr 15;19(8):e202502742. doi: 10.1002/cssc.202502742 (PMC13082860; doi:10.1002/cssc.202502742)
Supplement: Supplementary file 1 — Supplementary Material [file CSSC-19-e202502742-s001.pdf]

Supporting Information

Impact of Electron Transport Layers on Hysteresis and Performance of Ambient-processed Perovskite Solar Cells

*Qian Chen *[a], Abhinav K. Singh [ab], Hissah Alghatham [ab], Hongbo Mo [c], Feihong Li [c], Richard J. Curry [de], Laurie J Phillips [b], Amanda J. Hughes *[a]*

[a] Department of Materials, Design & Manufacturing Engineering, School of Engineering, University of Liverpool.

[b] Stephenson Institute for Renewable Energy, Department of Physics, University of Liverpool, Liverpool, L69 7ZF UK

[c] Department of Materials, School of Natural Science, The University of Manchester, Oxford Road, Manchester M13 9PL, UK

[d] Department of Electrical and Electronic Engineering, Photon Science Institute, University of Manchester, Manchester, M13 9PL, UK

[e] Department of Electrical and Electronic Engineering, University of Manchester, Manchester M13 9PL, UK

*Corresponding authors: qian.chen@liverpool.ac.uk, amanda.hughes2@liverpool.ac.uk

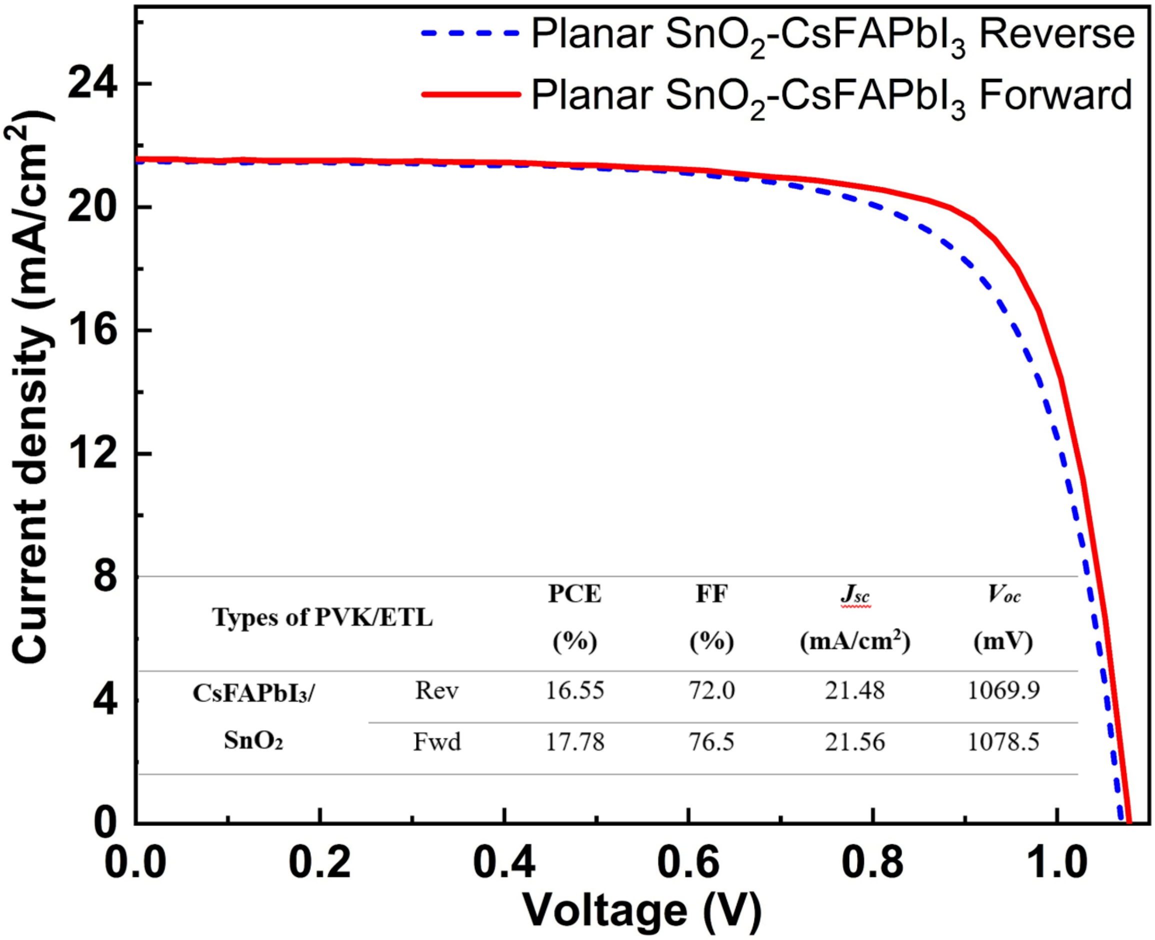

Types of PVK/ETL		PCE (%)	FF (%)	J_{sc} (mA/cm^2)	V_{oc} (mV)
$CsFAPbI_3$/ SnO_2	Rev	16.55	72.0	21.48	1069.9
	Fwd	17.78	76.5	21.56	1078.5

Figure S1 *J*–*V* characteristics of glovebox-processed PSCs based on the planar SnO_2-$CsFAPbI_3$.

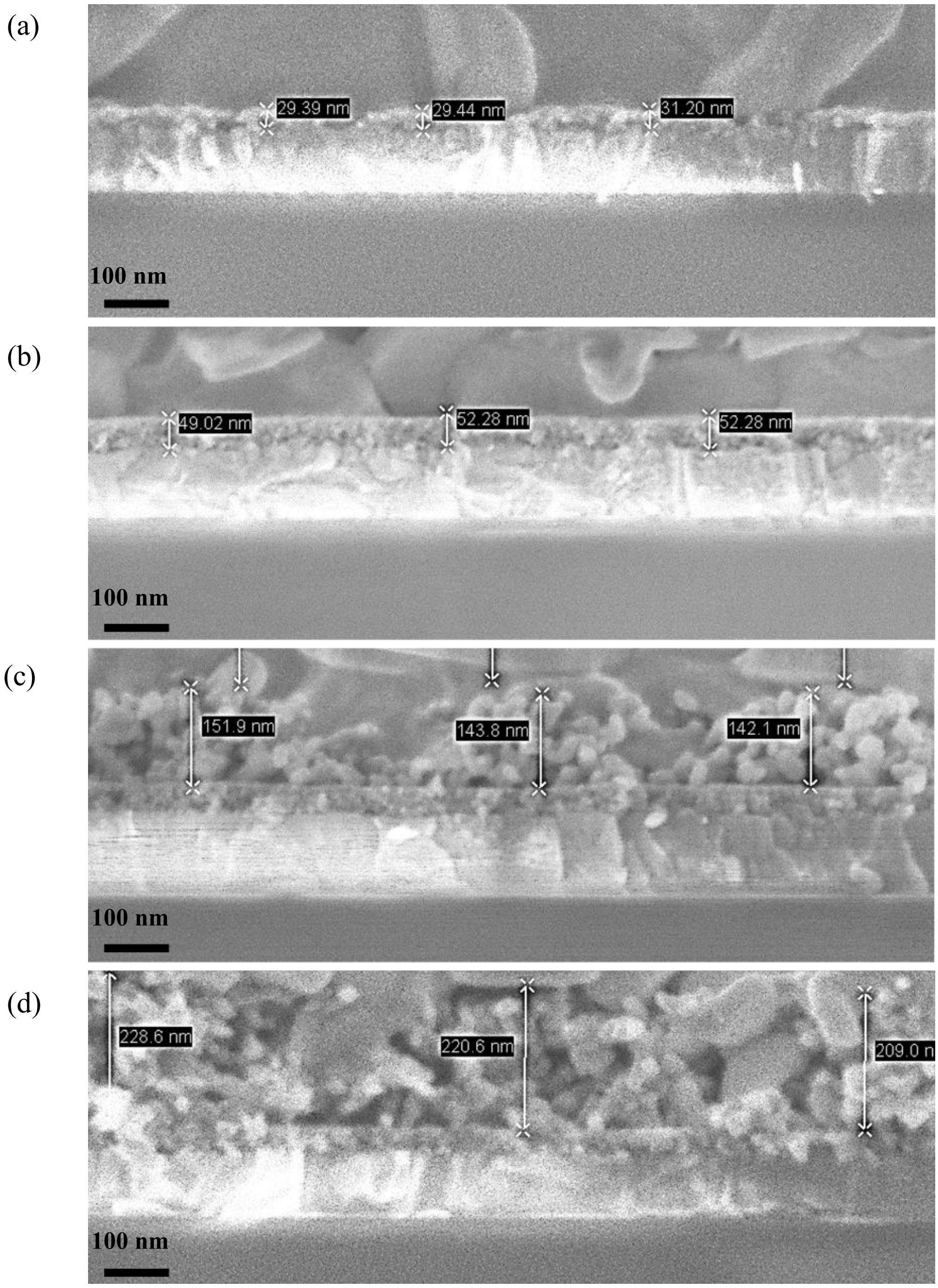

Figure S2 Cross-sectional SEM view of a) CP-30, b) CP-50, c) Meso-140 and d) Meso-220.

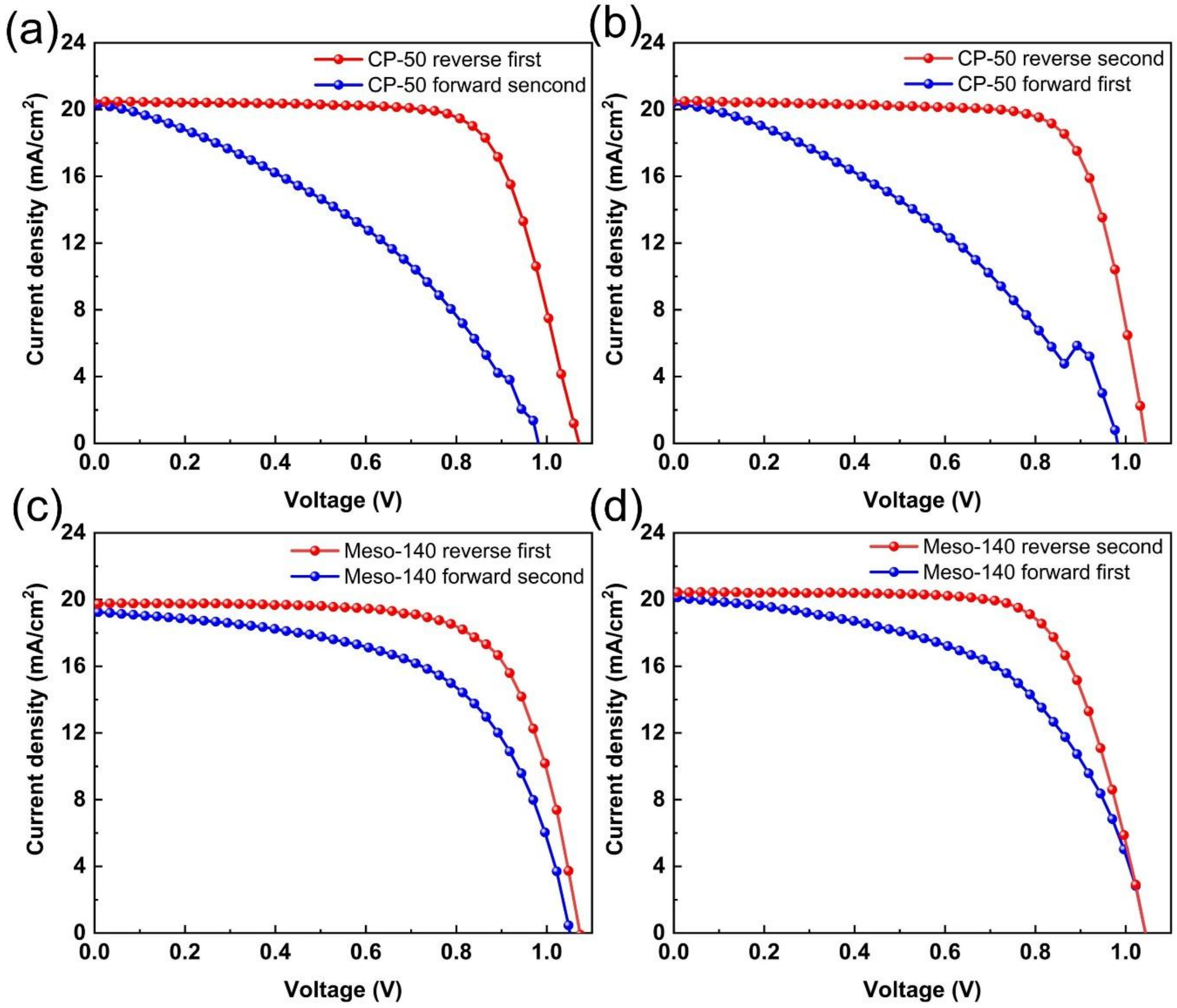

Figure S3 *J–V* characteristics of ambient-processed $MAPbI_3$ PSCs based on the (a) CP-50 with reverse scan first, (b) CP-50 with forward scan first, (c) Meso-140 with reverse scan first, and (d) Meso-140 with forward scan first.

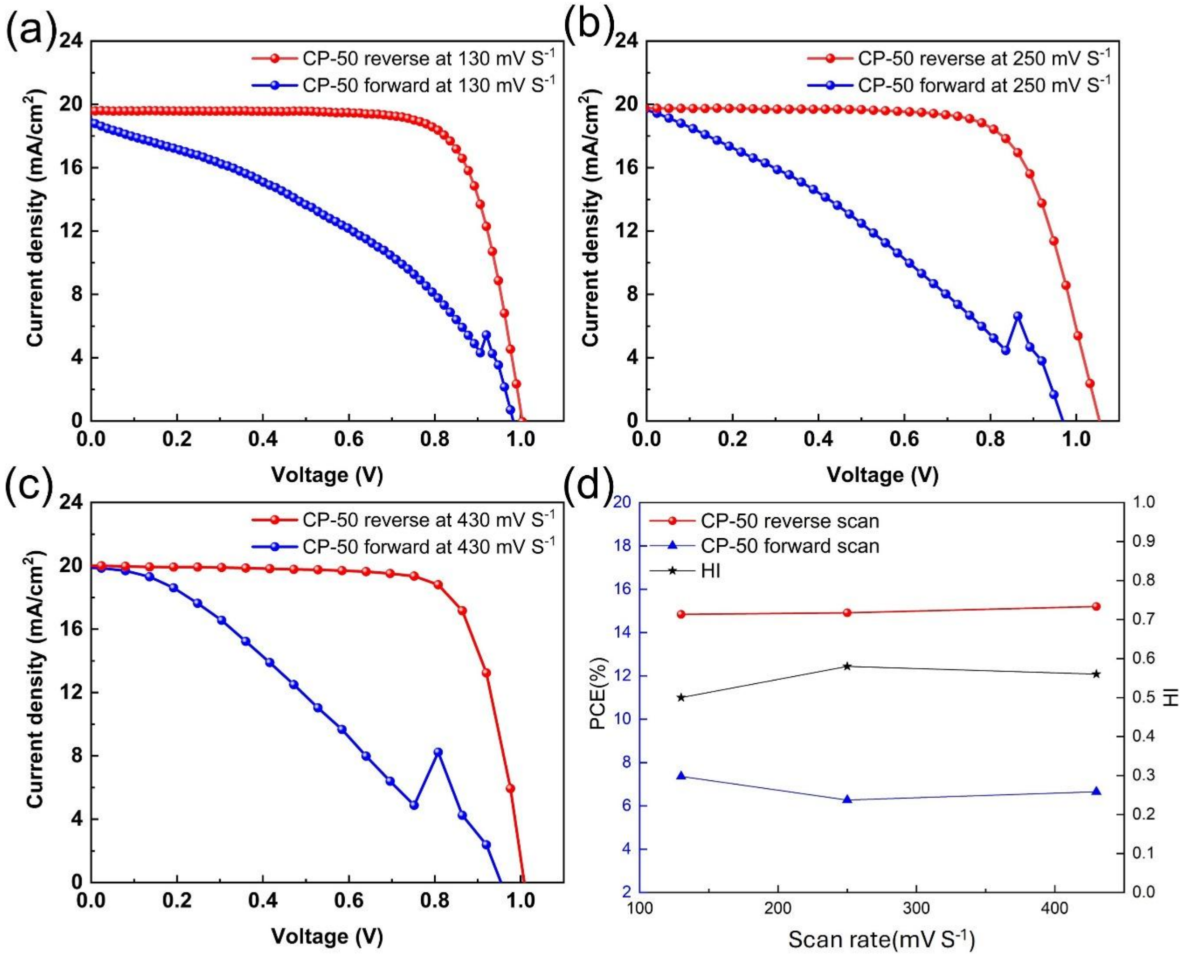

Figure S4 *J–V* characteristics of ambient-processed $MAPbI_3$ PSCs based on the CP-50 at the scan rates of (a) 130 mV s^{-1}, (b) 250 mV s^{-1}, and (c) 430 mV s^{-1}. (d) A summary of reverse-scan PCEs, forward-scan PCEs, and HI against various scan rates.

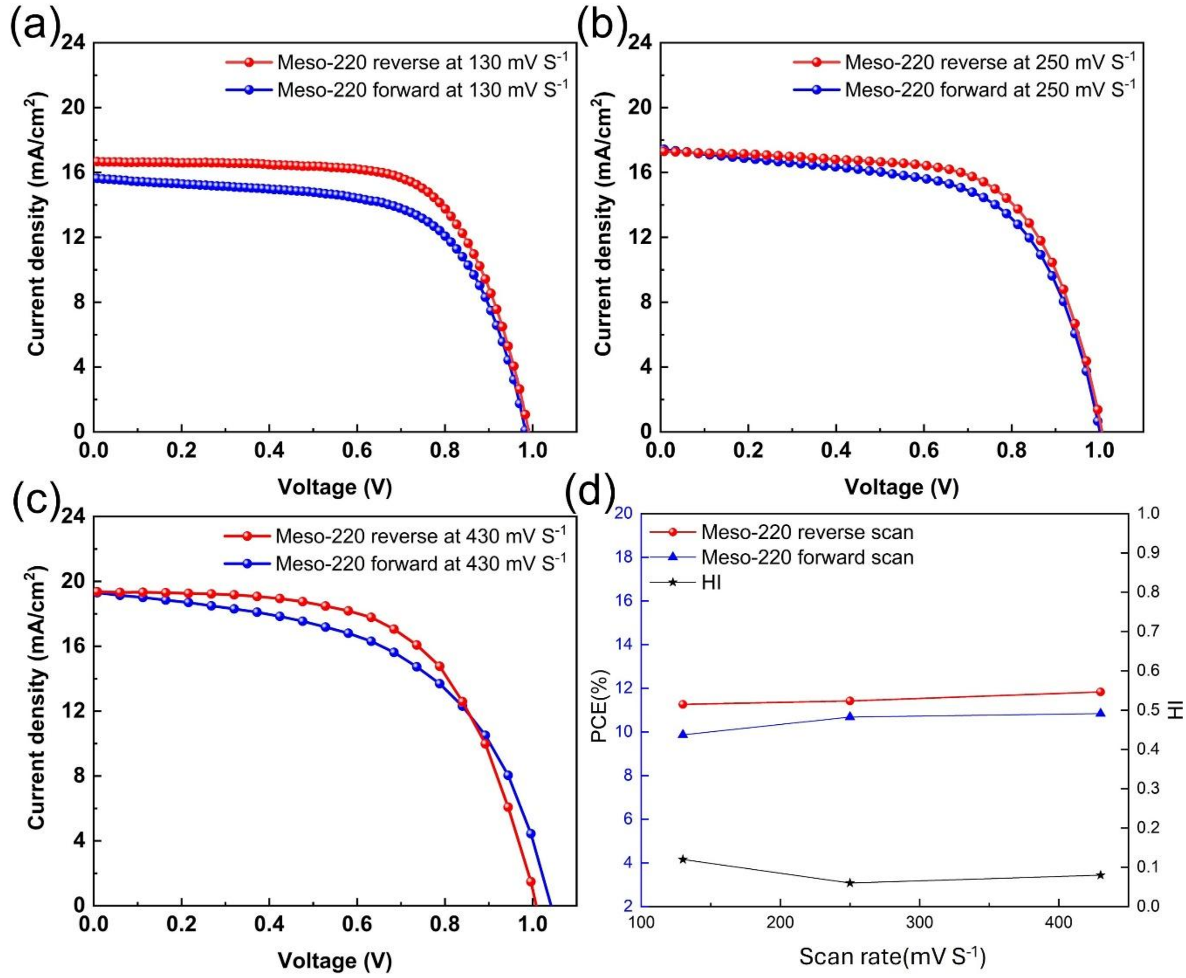

Figure S5 *J–V* characteristics of ambient-processed $MAPbI_3$ PSCs based on the Meso-220 at the scan rates of (a) 130 mV s^{-1}, (b) 250 mV s^{-1}, and (c) 430 mV s^{-1}. (d) A summary of reverse-scan PCEs, forward-scan PCEs, and HI against various scan rates.

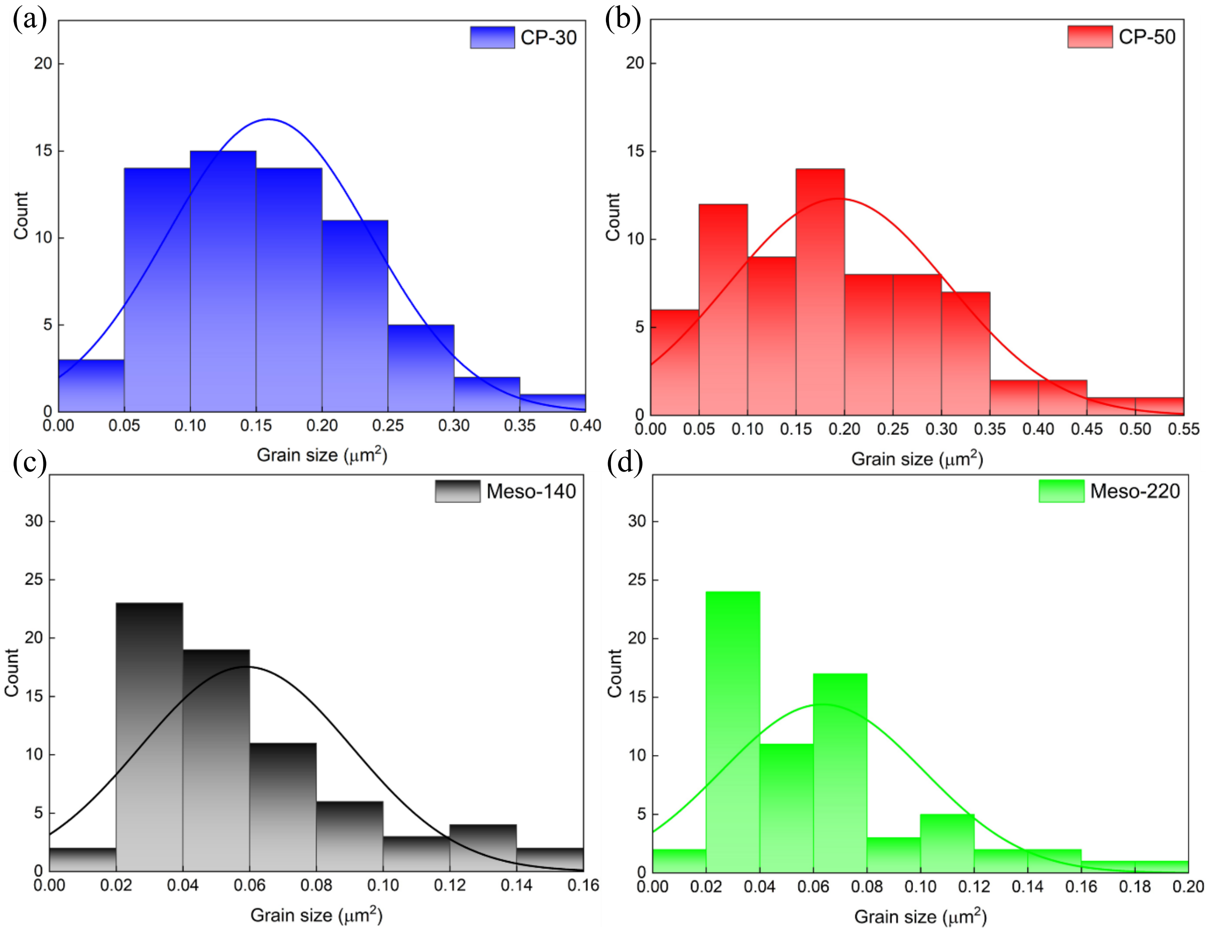

Figure S6 Grain size distribution for ambient-processed $MAPbI_3$ films based on the a) CP-30, b) CP-50, c) Meso-140 and d) Meso-220.

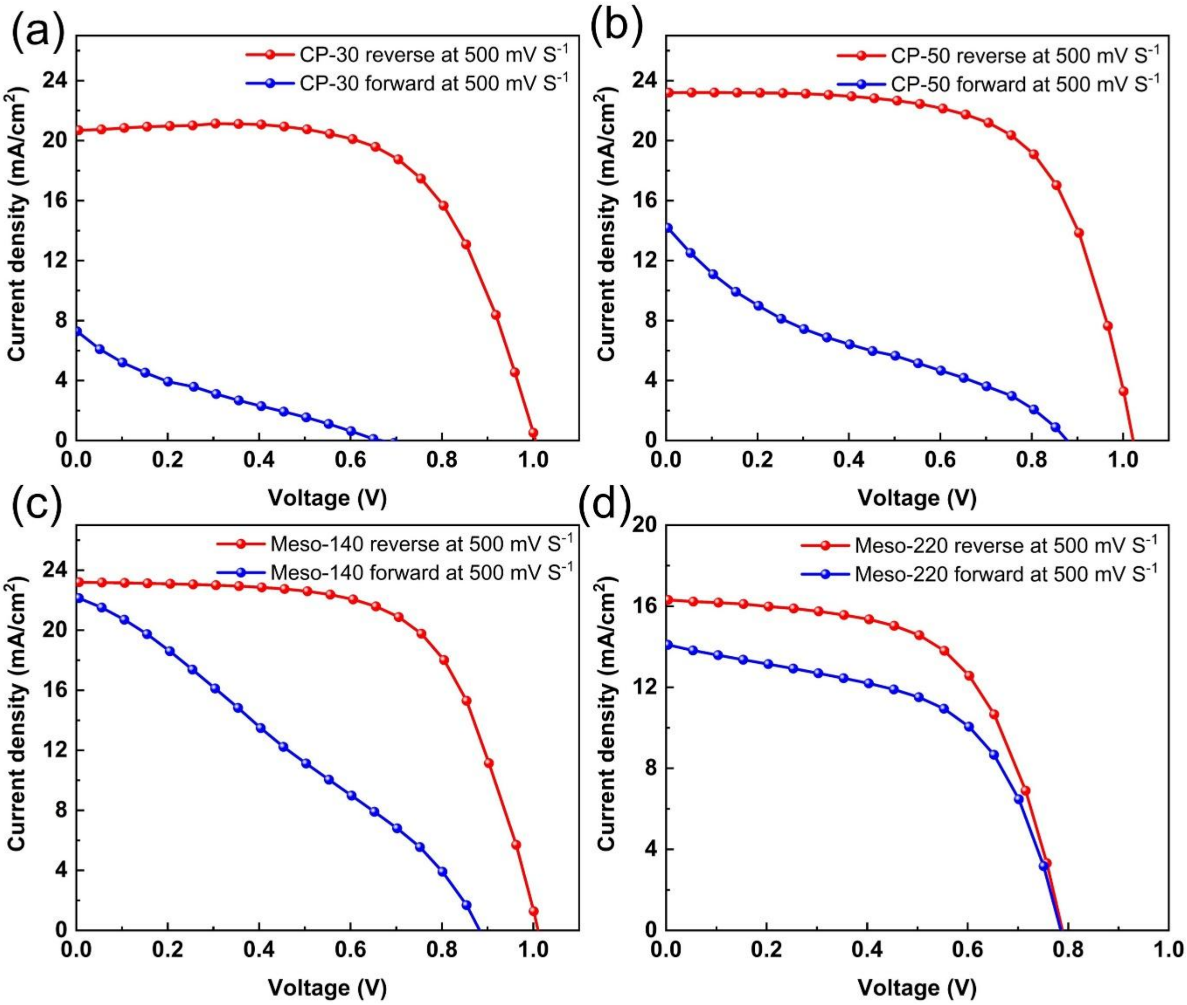

Figure S7 *J–V* characteristics of ambient-processed $MAPbI_3$ PSCs based on the (a) CP-50 fresh device, (b) CP-50 aged in ambient air for 9 days (c) Meso-140 fresh device, and (d) Meso-140 aged in ambient air for 9 days.

Table S1 Summary of photovoltaic parameters of the ambient-processed planar PSCs based on the TiO_2-$MAPbI_3$, TiO_2-$CsFAPbI_3$, SnO_2-$CsFAPbI_3$, and SnO_2-$Cs_2AgBiBr_6$

**Types of PVK/ETL**		**PCE (%)**	**FF (%)**	J_{sc} **(mA/cm²)**	V_{oc} **(mV)**	**HI**
$MAPbI_3/TiO_2$	Rev	14.02	69.3	19.81	1021.2	0.70
	Fwd	4.18	22.7	19.54	941.9	
$CsFAPbI_3/TiO_2$	Rev	17.16	71.4	23.53	1022.0	0.56
	Fwd	7.55	34.1	22.57	981.7	
$CsFAPbI_3/SnO_2$	Rev	17.76	74.5	23.19	1027.7	0.47
	Fwd	9.37	43.2	23.17	935.6	
$Cs_2AgBiBr_6/SnO_2$	Rev	0.73	64.2	1.03	1102.9	0.32
	Fwd	0.50	59.5	1.04	801.5	

Table S2 Summary of photovoltaic parameters of the ambient-processed $MAPbI_3$ PSCs based on the CP-30, CP-50, Meso-140, and Meso-220.

**Types of PVK/ETL**		**PCE (%)**	**FF (%)**	**J_{sc} (mA/cm^2)**	**V_{oc} (mV)**	**HI**
$MAPbI_3$/ CP-30	Rev	14.02	69.3	19.81	1021.2	0.70
	Fwd	4.18	22.7	19.54	941.9	
	Avg (Rev)	12.51±0.96	64.4±3.7	19.33±0.93	1006.3±20.2	
$MAPbI_3$/ CP-50	Rev	16.02	74.8	20.51	1044.2	0.52
	Fwd	7.73	38.7	20.32	981.6	
	Avg (Rev)	14.60±0.78	71.2±2.3	20.14±0.43	1017.8±40.1	
$MAPbI_3$/ Meso-140	Rev	15.67	72.4	20.72	1044.1	0.19
	Fwd	12.70	64.8	20.31	964.2	
	Avg (Rev)	14.53±1.10	69.8±2.7	19.95±0.77	1043.0±36.1	
$MAPbI_3$/ Meso-220	Rev	12.56	68.1	18.08	1021.4	0.09
	Fwd	11.43	62.4	18.06	1015.2	
	Avg (Rev)	11.88±0.92	66.5±2.6	18.14±1.32	986.2±37.2	

Table S3 Summary of TRPL results for the ambient-processed $MAPbI_3$ based on the CP-30, CP-50, Meso-140, and Meso-220.

	**T1**	**T2**	**Tavg**
cp-30	23.07	166.04	74.91
cp-50	23.68	95.43	37.16
meso-140	20.30	149.87	52.94
meso-220	23.24	125.52	41.44

Table S4 Summary of photovoltaic parameters of the fresh and aged ambient-processed PSCs devices based on the CP-50 and Meso-140.

**Types of PVK/ETL**		**PCE (%)**	**FF (%)**	J_{sc} **(mA/cm²)**	V_{oc} **(mV)**
$MAPbI_3$/ CP-50 Fresh	Rev	15.38	64.9	23.17	1022.9
	Fwd	2.84	21.9	14.71	880.1
$MAPbI_3$/ CP-50 after 9 days	Rev	13.10	59.37	22.42	983.9
	Fwd	0.61	5.98	12.73	807.5
$MAPbI_3$/ Meso-140 Fresh	Rev	14.93	63.7	23.19	1010.0
	Fwd	5.59	29.3	21.65	882.6
$MAPbI_3$/ Meso-140 after 9 days	Rev	15.03	63.5	23.56	1005.0
	Fwd	2.51	14.1	20.02	891.9

Table S5 Summary of photovoltaic parameters of the ambient-processed $CsFAPbI_3$ and $Cs_2AgBiBr_6$ PSCs based on the different ETLs

**Types of PVK/ETL**		**PCE (%)**	**FF (%)**	J_{sc} **(mA/cm²)**	V_{oc} **(mV)**	**HI**
$CsFAPbI_3$/CP-50	Rev	17.16	71.4	23.53	1022.0	0.56
	Fwd	7.55	34.1	22.57	981.7	
$CsFAPbI_3/SnO_2$	Rev	17.76	74.5	23.19	1027.7	0.47
	Fwd	9.37	43.2	23.17	935.6	
$CsFAPbI_3$/Meso-140	Rev	17.40	71.9	23.25	1041.6	0.38
	Fwd	10.77	51.1	22.58	933.6	
$Cs_2AgBiBr_6/SnO_2$	Rev	0.73	64.2	1.03	1102.9	0.32
	Fwd	0.50	59.5	1.04	801.5	
$Cs_2AgBiBr_6$/Meso-140	Rev	0.71	63.8	1.09	1020.3	0.08
	Fwd	0.65	61.5	1.01	1040.1	
